# Composition of the Intestinal Microbiota Determines the Outcome of Virus-Triggered Colitis in Mice

**DOI:** 10.3389/fimmu.2019.01708

**Published:** 2019-07-23

**Authors:** Silvia Bolsega, Marijana Basic, Anna Smoczek, Manuela Buettner, Claudia Eberl, Daniel Ahrens, Kodwo Appoh Odum, Bärbel Stecher, Andre Bleich

**Affiliations:** ^1^Hannover Medical School, Institute for Laboratory Animal Science, Hanover, Germany; ^2^Faculty of Medicine, Max von Pettenkofer Institute of Hygiene and Medical Microbiology, LMU Munich, Munich, Germany; ^3^German Center of Infection Research (DZIF), Partner Site Munich, Munich, Germany

**Keywords:** gnotobiotic models, intestinal microbiota, ASF, Oligo-MM^12^, MNV, colitis, *Il10*-deficient mice, SFB

## Abstract

The intestinal microbiota is a complex ecosystem implicated in host health and disease. Inflammatory bowel disease (IBD) is a multifactorial chronic disorder of the gastrointestinal mucosa. Even though the exact mechanisms are still unknown, the intestinal microbiota is crucial in IBD development. We previously showed that murine norovirus (MNV) induces colitis in the *Il10*-deficient (*Il10*^−/−^) mouse model of IBD in a microbiota-dependent manner. Thus, in this study we analyzed whether distinct minimal bacterial consortia influence the outcome of MNV-triggered colitis in *Il10*^−/−^ mice. Gnotobiotic *Il10*^−/−^ mice associated with Oligo-Mouse-Microbiota 12 (OMM^12^) or Altered Schaedler Flora (ASF) developed little to no inflammatory lesions in the colon and cecum. MNV infection exacerbated colitis severity only in ASF-colonized mice, but not in those associated with OMM^12^. Four weeks after MNV infection, inflammatory lesions in ASF-colonized *Il10*^−/−^ mice were characterized by epithelial hyperplasia, infiltration of inflammatory cells, and increased barrier permeability. Co-colonization of ASF-colonized *Il10*^−/−^ mice with segmented filamentous bacteria (SFB) abolished MNV-induced colitis, whereas histopathological scores in SFB-OMM^12^-co-colonized mice stayed unchanged. Moreover, SFB only colonized mice associated with ASF. The SFB-mediated protective effects in ASF-colonized mice involved enhanced activation of intestinal barrier defense mechanisms and mucosal immune responses in the chronic and acute phase of MNV infection. SFB colonization strengthened intestinal barrier function by increasing expression of tight junction proteins, antimicrobial peptides and mucus. Furthermore, SFB colonization enhanced the expression of pro-inflammatory cytokines such as *Tnf*α, *Il1*β, and *Il12a*, as well as the expression of the regulatory cytokine *Tgf*β. Altogether, our results showed that MNV-triggered colitis depends on the microbial context.

## Introduction

The intestinal microbiota is a highly complex ecosystem dominated by four bacterial phyla, namely, *Bacteroidetes, Firmicutes, Actinobacteria*, and *Proteobacteria* (1, 2). Its pronounced influence on host health and disease was corroborated over the last decades by a multitude of publications. The gut microbiota is involved in many physiological processes such as synthesis of vitamins, production of short chain fatty acids (SCFA), bioconversion of complex molecules, degradation of xenobiotic substances, and also in the development and maturation of the mucosal immune system (3, 4). On the other hand, the intestinal microbiota is also implicated in the development of many human disorders such as inflammatory, autoimmune and metabolic diseases, as well as tumorigenesis (5–8). However, it is still unclear whether identified changes in microbiota composition and function are a cause or a consequence of disease, since most of studies are based on associations and lack fundamental proof of causality. In addition, the composition of the intestinal microbiota is complex and non-defined and thus causal microbial effects cannot be appropriately addressed (9–11). Thus, this emphasizes the need to perform mechanistic studies to understand causal interrelations between the microbiome and the host in health and disease. Gnotobiotic animal models represent a powerful tool for investigating functional effects of host-microbe and microbe-microbe interactions (12). These models include germ-free (GF) animals that are devoid of all other living organisms and animals that are colonized with known microorganisms. The possibility to colonize GF animals with defined microorganisms allows us to analyze complex host-microbiota interactions mechanistically in a simplified way. Utilizing minimal bacterial consortia in animal models reduces the microbiome complexity on a manageable level and supports studies that can evaluate the impact of particular microorganisms on the host physiology. Furthermore, these approaches can contribute to the development of novel therapeutic or prophylactic strategies that would allow non-invasive modulation of the intestinal microbiota.

Inflammatory bowel disease (IBD) is a multifactorial chronic relapsing inflammatory disorder of the gastrointestinal tract and has two main forms—Crohn's disease and ulcerative colitis. The intestinal microbiota was shown to be crucial for the development of IBD, while genetic susceptibility and environmental factors can also play an important role (13, 14). The exact mechanisms and involvement of commensal microbiota in IBD development are still not fully understood. Hence, we generated a gnotobiotic model of experimental IBD to dissect the underlying mechanisms of the cross-talk between particular microbes and the host response during IBD development. Our experimental IBD model consists of three defined factors: a susceptible host (GF *Il10*-deficient mice), defined bacterial microbiomes [Altered Schaedler Flora (ASF) or Oligo-Mouse-Microbiota 12 (OMM^12^)], and murine norovirus (MNV) infection as a specific trigger. *Il10*-deficient (*Il10*^−/−^) mice lack regulatory cytokine interleukin 10 (IL10) and spontaneously develop intestinal inflammation (15). The genetic background of *Il10*^−/−^ mice is an important factor that determines colitis susceptibility. For example, *Il10*^−/−^ mice on a C3H/HeJBir or 129/SvEv background show higher colitis susceptibility than *Il10*^−/−^ mice on C57BL/6J background (16, 17). However, the intestinal microbiota was shown to be essential for disease development, as GF *Il10*^−/−^ mice do not develop colitis (18, 19). The composition of the intestinal microbiota can alter colitis severity of *Il10*^−/−^ mice (20, 21). Previously, we have shown that MNV infection triggers colitis in *Il10*^−/−^ mice in a microbiota-dependent manner (22).

In this study, GF *Il10*^−/−^ mice were colonized with two different defined minimal bacterial consortia, ASF and OMM^12^. ASF is a well-established model community consisting of eight bacterial species. These bacterial species were isolated from the mouse gut and belong to three bacterial phyla: *Bacteroidetes, Firmicutes*, and *Deferribacteres* (Table 1). The ASF consortium can be stably maintained for generations under gnotobiotic conditions (23, 24). This minimal microbiota is widely used in biomedical research including studies addressing perturbations of the microbiota composition upon infection and evaluating the impact of particular microorganisms on the host immune system (24). Moreover, this consortium is devoid of pathobionts and does not cause overt mucosal inflammation (25–27). The second minimal microbiota used in this study is a defined bacterial consortium OMM^12^ (28). This model community contains 12 mouse enteric microbiota-derived bacterial species that represent five major bacterial phyla including *Bacteroidetes, Firmicutes, Verrucomicrobia, Proteobacteria*, and *Actinobacteria* (Table 1) (28, 29). This defined bacterial community was designed to analyze mechanisms of colonization resistance to enteric infections (28). Additionally, all OMM^12^ bacterial strains are included in the publicly accessible catalog, the mouse intestinal bacterial collection (miBC), which makes them attractive for application in mechanistic microbiome studies (30). In this study defined bacterial microbiomes were additionally modulated by segmented filamentous bacteria (SFB). SFB are gram-positive, spore-forming commensal bacteria of the *Clostridiaceae* family that are found in the gastrointestinal tract of several different species. These bacteria grow attached to epithelial cells and potently stimulate the host's mucosal immune system, especially IL17-mediated immune responses (31, 32). Due to their strong immunomodulatory functions, SFB can cause beneficial or adverse effects on the host physiology (32).

**Table 1:** Taxonomic classification of ASF and OMM^12^ bacterial strains.

**ASF**		**Phylum**	**OMM**^****12****^	
*Clostridium sp*.	ASF356	*Firmicutes*	I49	*Lactobacillus reuteri*
*Lactobacillus acidophilus*	ASF360		I46	*Clostridium innocuum*
*Lactobacillus murinus*	ASF361		YL32	*Clostridium clostridioforme*
*Eubacterium plexicaudatum*	ASF492		YL31	*Flavonifractor plautii*
*Pseudoflavonifractor sp*.	ASF500		YL58	*Blautia coccoides*
*Clostridium sp*.	ASF502		KB1	*Enterococcus faecalis*
			KB18	*Acutalibacter muris*
*Parabacteroidetes goldsteinii*	ASF519	*Bacteroidetes*	I48	*Bacteroides caecimuris*
			YL27	*Muribaculum intestinale*
*Mucispirillum schaedleri*	ASF457	*Deferribacteres*		
		*Proteobacteria*	YL45	*Turicimonas muris*
		*Actinobacteria*	YL2	*Bifidobacterium longum subsp. animalis*
		*Verrucomicrobia*	YL44	*Akkermansia muciniphila*

*ASF, Altered Schaedler Flora; OMM^12^, Oligo-Mouse-Microbiota 12*.

Overall, the aim of this study was to assess whether minimal bacterial consortia influence the outcome of MNV-triggered colitis in the *Il10*^−/−^ mouse model of IBD and how this phenotype is modulated by specific commensals.

## Materials and Methods

### Mice

Germ-free (GF) male and female C57BL/6J.129P2-*Il10*^*tm*1*Cgn*^/ JZtm (B6-*Il10*^−/−^), C3H/HeJBir.129P2-*Il10*^*tm*1*Cgn*^/JZtm (C3H-*Il10*^−/−^), C57BL/6;129Sv-*Rag2*^*tm*1*Fwa*^/Ztm (B6-*Rag2*^−/−^), and gnotobiotic C57BL/6JZtm^OMM12^, C.B-Igh1^b^/IcrTac^ASF^ (C.B-17^ASF^) and NOD/LtSz.CB17-*Prkdc*^scid^/JZtm^SFB^ (NOD-scid^SFB^) mice were obtained from the Central Animal Facility (Hannover Medical School, Hanover, Germany). Breeding of gnotobiotic animals was performed in plastic film isolators (Metall+Plastik GmbH, Radolfzell-Stahringen, Germany) located in a room with a controlled environment and 12 h light/dark cycles. For experiments, mice were maintained in airtight cages with positive pressure (IsoCage P, Tecniplast Deutschland GmbH, Bavaria, Germany) to keep their gnotobiotic status. Mice received pelleted 50 kGy gamma-irradiated feed and autoclaved water *ad libitum*. Mice were sacrificed by CO_2_ inhalation followed by exsanguination at 12 weeks of age (chronic MNV infection) or 8 weeks of age (acute MNV infection). To collect enough tissue for all analyses, chronic MNV infection and colonization experiments in B6-*Il10*^−/−^ mice were divided in two cohorts. From one cohort, samples for histology (small intestine, cecum and colon) were collected. From the second cohort, tissue samples for gene expression, western blot, immunofluorescence, and fluorescence *in situ* hybridization analyses were harvested. The colon tissue was sampled starting from the proximal part in this order: tissue for gene expression (ca. 0.5 cm), western blot (ca. 0.5 cm), immunohistology (ca. 1.5 cm), and fluorescence *in situ* hybridization (ca. 1.5 cm). For each analysis, section-matched tissue was used. Each cohort consisted of at least 5 mice per group and each experiment was performed one, two or three times. Gnotobiotic animals bred at the Central Animal Facility were monitored according to recommendations for maintaining gnotobiotic colonies (33) and FELASA recommendations (34) and were proven to be free of contaminants or infection with common murine pathogens. Furthermore, all experimental groups were screened for the presence of microbial contaminants at the end of the experiment. Animals confirmed to have unexpected microbes were omitted from analyses. The presence of contaminating bacteria was controlled in DNA isolated from feces by 16S rRNA gene sequencing analysis. Furthermore, to rule out contamination with MNV, non-infected groups were randomly tested for the presence of MNV.

### Colonization of Germ-Free Mice

Four week old GF B6-*Il10*^−/−^ and C3H-*Il10*^−/−^ mice were colonized with one of the two minimal consortia (ASF or OMM^12^) via co-housing for 4 weeks with gnotobiotic donor animals (C57BL/6JZtm^OMM12^ and C.B-17^ASF^). Juvenile GF B6-*Rag2*^−/−^ mice were also associated with OMM^12^ via co-housing over a period of 4 weeks. To generate SFB gut content aliquots for inoculation, three SFB monoassociated mice (NOD-scid^SFB^) were sacrificed. Subsequently, ileum and cecum were harvested and placed in a cold sterile petri dish. The lumen was flushed with sterile Luria-Bertani media (1:2/ intestinal content:media). Shortly after larger fecal particles settled, intestinal content was transferred into cryotubes. Per cryotube, 850 μL of intestinal content was mixed with 150 μL glycerol and stored at −80°C until needed. From these aliquots, mice were colonized with SFB via oral gavage of 50 μL of intestinal content on 2 consecutive days.

### Virus Infection

MNV strain Hannover1/2007/DEU was used for infection (22). MNV was propagated on RAW264.7 cells as described previously (35). Eight week old germ-free or gnotobiotic B6-*Il10*^−/−^ and C3H-*Il10*^−/−^ mice colonized with ASF, OMM^12^, and/or SFB were infected with 100 μl virus suspension (5 × 10^3^/ml TCID_50_) via oral gavage. Control groups were left untreated.

### Polymerase Chain Reaction (PCR)

DNA extraction from feces of ASF-colonized mice was performed using the PSP^®^ Spin Stool DNA Kit (Stratec Molecular GmbH, Berlin, Germany) following the manufacturer's instructions. To detect the ASF consortium, species-specific forward (FW) and reverse (RV) primers synthesized by Eurofins (Eurofins Genomics, Ebersberg, Germany) were used to detect 16S rRNA genes (Table 2). DNA was amplified using OneTaq^®^ Hot Start 2X Master Mix with Standard Buffer (New England Biolabs, Ipswich, MA, USA) following the manufacturer's protocol. The thermocycling conditions included: (i) an initial denaturation step of 30 s at 94°C; (ii) 30 cycles of 30 s at 94°C, 45 s at 60°C (annealing temperature) and 1 min/kb at 68°C; and (iii) a final elongation step of 5 min at 68°C. PCR products were subjected to capillary electrophoresis using QIAxcel Advanced System (Qiagen, Hilden, Germany).

**Table 2:** List of PCR primers for detection of ASF members.

**Strain**	**Name**	**Primer sequence^a^ (5^′^ 3^′^)**	**Amplicon size (bp)**	**GenBank accession number^b^**
ASF356	*Clostridium sp*.	FW—CGGTGACTAATACCGCATACGG	417	AF157052
		RV—CCTTGCCGCCTACTCTCCC		
ASF360	*Lactobacillus acidophilus*	FW—CTTCGGTGATGACGCTGG	131	AF157050
		RV—GCAATAGCCATGCAGCTATTGTTG		
ASF361	*Lactobacillus murinus*	FW—GCAATGATGCGTAGCCGAAC	182	AF157049
		RV—GCACTTTCTTCTCTAACAACAGGG		
ASF457	*Mucispirillum schaedleri*	FW—CCGAAAGGTGAGCTAATGCCGG	95	AF157055
		RV—GGGACGCGAGTCCATCTTTC		
ASF492	*Eubacterium plexicaudatum*	FW—CTGCGGAATTCCTTCGGGG	167	AF157054
		RV—CCCATACCACCGGAGTTTTC		
ASF500	*Pseudoflavonifractor sp*.	FW—GTCGCATGGCACTGGACATC	285	AF157051
		RV—CCTCAGGTACCGTCACTTGCTTC		
ASF502	*Clostridium sp*.	FW—CGGTACCGCATGGTACAGAGG	427	AF157053
		RV—CAATGCAATTCCGGGGTTGG		
ASF519	*Parabacteroidetes goldsteinii*	FW—CACAGTAAGCGGCACAGCG	429	AF157056
		RV—CCGCTCACACGGTAGCTG		

*FW, forward; RV, reverse*.

a *Primer design was described by Sarma-Rupavtarm et al. (36)*.

b *GenBank accession numbers were published by Dewhirst et al. (23)*.

### Quantitative Real-Time PCR (qPCR)

For gene expression analyses, the proximal colon was collected. The colon tissue was flushed with sterile PBS, snap frozen in liquid nitrogen, and kept at −80°C until further processing. Total RNA was extracted from the proximal colon tissue using the RNeasy Kit (Qiagen, Hilden, Germany), including an additional step of on-column DNase digestion (RNase-Free DNase Set, Qiagen, Hilden, Germany). The cDNA synthesis was carried out using the QuantiTect Reverse Transcription Kit (Qiagen, Hilden, Germany) according to the manufacturer's recommendations. The cDNA samples were diluted 1:10 using HPLC grade water (J. T. Baker, Deventer, Netherlands). The qPCR was performed using QuantiTect Primer Assays (Qiagen) or TaqMan^®^ Gene Expression Assays (ThermoFisher Scientific) as recommended by the manufacturer (Table 3). Beta actin was used as a reference gene in both assays. Detection was performed with the StepOnePlus™ or QuantStudio 6 Flex Real-Time PCR System (Applied Biosystems, Weiterstadt, Germany) using the Fast SYBR Green^®^ Master Mix or TaqMan^®^ Fast Advanced Master Mix according to the manufacturer's instruction. All reactions were run in triplicate. The thermocycling conditions for SYBR Green^®^ chemistry included: (i) a polymerase activation step of 20 s at 95°C; and (ii) 40 cycles of 3 s at 95°C and 30 s at 60°C (annealing and elongation step). The amplified PCR product was verified by melting curve analysis (for SYBR Green^®^ chemistry). The thermocycling conditions for TaqMan^®^ chemistry were: (i) an incubation step of 2 min at 50°C; (ii) a polymerase activation step of 2 min at 95°C; and (iii) 40 cycles of 1 s at 95°C and 20 s at 60°C (annealing and elongation step). Relative gene expression was calculated using the 2^−ΔCt^ method.

**Table 3:** List of qPCR primers used for gene expression analyses.

**Assay chemistry**	**Gene**	**Name**	**Assay number**
SYBR^®^ Green	*Cldn4*	Claudin 4	Mm_Cldn4_1_SG
	*Cldn8*	Claudin 8	Mm_Cldn8_1_SG
	*Muc2*	Mucin 2	Mm_Muc2_2_SG
	*Defb2*	ß-defensin 2	Mm_Defb2_2_SG
	*Ifnl2*	Interferon lambda 2	Mm_Ifnl2_1_SG
	*Actb*	Beta actin	Mm_Actb_2_SG
TaqMan^®^	*Tnfα*	Tumor necrosis factor alpha	Mm_00443258_m1
	*Ifnγ*	Interferon gamma	Mm_01168134_m1
	*Il1β*	Interleukin 1 beta	Mm_00434228_m1
	*Il12a*	Interleukin 12a	Mm_00434169_m1
	*Il17a*	Interleukin 17a	Mm_00439618_m1
	*Reg3γ*	Regenerating islet-derived protein 3 gamma	Mm_00441127_m1
	*Tgfβ*	Transforming growth factor beta	Mm_01178820_m1
	*Mmp7*	Matrix metallopeptidase 7	Mm_00487724_m1
	*Foxp3*	Forkhead box P3	Mm_00475162_m1
	*Actb*	Beta actin	Mm_00607939_s1

The absolute quantification using a standard curve performed with the QuantStudio 6 Flex Real-Time PCR System was used to determine MNV and SFB gene copy numbers/μL in the colon tissue or feces. For MNV quantification, the proximal colon tissue was harvested and prepared as described above including total RNA and cDNA isolation. MNV load was determined using a TaqMan assay with an MNV-specific primer/probe set (Table 4) and the TaqMan^®^ Fast Advanced Master Mix as recommended by the manufacturer. The thermocycling conditions for MNV detection included: (i) a polymerase activation step of 20 s at 95°C; and (ii) 40 cycles of 1 s at 95°C and 20 s at 60°C (annealing and elongation step). For SFB quantification, DNA from one fecal pellet per animal was isolated using the PSP^®^ Spin Stool DNA Kit (Stratec Molecular GmbH) according to the manufacturer's instructions, including an initial bead-beating step using zirconia beads. Isolated DNA was diluted to a final concentration of 25 ng/μL using HPLC grade water (J. T. Baker) and stored at −20°C until further processing. SFB 16S rRNA gene copy numbers were determined using a TaqMan assay with an SFB-specific primer/probe set (Table 4) and Taqman^®^ Universal Master Mix according to the manufacturer's instruction. The thermocycling conditions for SFB detection were: (i) an incubation step of 2 min at 50°C; (ii) a polymerase activation step of 10 min at 95°C; and (iii) 40 cycles of 15 s at 95°C and 1 min at 60°C (annealing and elongation step). Both qPCR assays were performed using 900 nM of each primer and 200 nM of a specific probe. The qPCR standards for quantifying MNV and SFB abundance were generated using plasmids containing MNV or SFB amplicon products generated with specific primers listed in Table 4. The MNV and SFB amplicon products were cloned into a pSC-A-amp/kan vector using the StrataClone PCR Cloning Kit (Agilent Technologies, La Jolla, CA, USA) following manufacturer's instructions. The plasmid DNA was purified by NucleoSpin^®^ Plasmid Kit followed by NucleoBond^®^ Xtra Maxi Kit (Macherey-Nagel GmbH & Co. KG, Duren, Germany) according to the manufacturer's protocol. Standard curves for MNV and SFB were generated using a 10-fold dilution series of the plasmids ranging from 1 to 10^4^ copies/μL for MNV and 1 to 10^7^ copies/μL for SFB. The detection of 16S rRNA gene copy numbers of particular OMM^12^ members was performed as described by Brugiroux et al. (28).

**Table 4:** QPCR primers and TaqMan probes for MNV and SFB quantification.

**Species**	**MNV**	**SFB**
Clone	F8	Z4077
FW primer sequence (5′ 3′)	ACGCCACCGATCTGTTCTG	ACGCTGAGGCATGAGAGCAT
RV primer sequence (5′ 3′)	AGACTGCTGAGCGTTCCTG	GACGGCACGGATTGTTATTCA
TaqMan probe sequence (5′ 3′)	FAM-CATCCATTGTTCCAAAGCGCACCCAGC -BHQ1	FAM-CTGGTAGTCCATGCTGTAAACGATGGGTACTAGG-BHQ1
Amplicon size (bp)	77	108

*MNV, Murine norovirus; SFB, Segmented filamentous bacteria; FW, forward; RV, reverse*.

### Histology

The small intestine, cecum, and colon were collected and fixed in neutral buffered 4% formalin. Subsequently, samples were dehydrated, embedded in paraffin, sectioned at 3 μm, and stained with hematoxylin and eosin (H&E). H&E stained cecum and colon sections were scored as described previously (37). Briefly, histopathological lesions were scored blindly for ulceration, hyperplasia, severity, and the involved area. Each parameter was graded from 0 (physiological) to 3 (severe changes) and added in a total score from 0 to 12. Colon sections were scored separately for the proximal, middle, and distal part. A total colon score was calculated by adding all three colon sections (maximal score 36).

### Immunofluorescence

Immunofluorescence staining for CD3 and CD45R was performed on formalin-fixed paraffin-embedded colon tissue sections. Sections were deparaffinized using xylol and rehydrated using decreasing concentrations of ethanol (100, 95, and 70%) followed by a short wash in distilled water. Heat-induced antigen retrieval was performed in citrate-based buffer (Target Retrieval Solution, Agilent Dako, Santa Clara, CA, USA) in 700 W microwave. Sections were blocked and permeabilized in PBS containing 10% horse serum and 0.1% Triton X-100 for 1 h at room temperature followed by overnight incubation at 4°C with either rabbit anti-CD3 monoclonal antibody (1:50; clone SP7, Abcam, Cambridge, UK) or rat anti-CD45R monoclonal antibody (1:200; clone RA3-6B2, Abcam). After three 5 min washes in PBS, sections were incubated for 2 h at room temperature with either DyLight^®^594 conjugated donkey anti-rabbit polyclonal secondary antibody (1:500, Abcam) or Alexa Fluor 555 conjugated goat anti-rat polyclonal antibody (1:500, Invitrogen, CA, USA). Immunofluorescence staining for mucin 2 was performed on Carnoy's solution (60% absolute ethanol, 30% chloroform, and 10% acetic acid) fixed and paraffin embedded colon tissue sections using rabbit anti-mucin 2 polyclonal primary antibody (1:100; antibodies-online GmbH, Aachen, Germany) and DyLight^®^594 conjugated donkey anti-rabbit polyclonal secondary antibody (1:250; Abcam). Nuclear counterstaining was performed with a mounting medium containing DAPI (Vectashield, Vector Laboratories, USA). Stained tissue sections were examined using the Zeiss Axioskop 40 microscope (Carl Zeiss Microscopy GmbH, Göttingen, Germany) connected to an AxioCam MRm (Carl Zeiss). For analyses, the number of CD3+ or CD45R+ cells was determined by counting stained cells per visual field (ten fields per slide). Mucin 2 staining was analyzed by measuring the mucus layer thickness using Zeiss ZEN blue software (seven animals per group, six images per animal, and five measurements per image). Mean value of five measurements per image was generated and data were presented as six technical replicates per animal. All images were taken and scored blindly.

### Fluorescence *in situ* Hybridization (FISH)

The small intestine and colon were fixed in Carnoy's solution overnight, embedded in paraffin, and sectioned at 3 μm. After deparaffinization, antigen retrieval was performed with lysis buffer (20 mM Tris, 2 mM EDTA, and 1.2% Triton-X-100 solution) containing lysozyme (40 mg/ml; Merck KGaA, Darmstadt, Germany) at 37°C for 45 min. Slides were subsequently treated with pre-warmed (50°C) hybridization buffer (0.9 M NaCl, 0.05% SDS, and 20 mM Tris-HCl at pH 7.4) containing 0.5 pmol/μl of the EUB338 probe (5′ - Cy3-GCT GCC TCC CGT AGG AGT - 3′) (38) and the SFB probe (5′ - Cy5-GGG TAC TTA TTG CGT TTG CGA CGG CAC - 3′). After incubation at 50°C for 3 h, the buffer was replaced by pre-warmed washing solution (0.9 M NaCl, 0.006% SDS, and 20 mM Tris-HCl at pH 7.4). The slides were covered with a mounting medium containing DAPI and examined as described above.

### Western Blot

Western blot analyses were performed as described previously (39). Briefly, total proteins were extracted from colon tissue and measured by Bradford Assay using Biorad-Dye-Reagent-Concentrate (Bio-Rad, Hercules, CA, USA) on the Victor^TM^ X3 reader (Perkin Elmer, Waltham, MA, USA). One hundred microgram proteins per sample were separated by sodium dodecyl sulfate polyacrylamide gel electrophoresis on 15% gels and transferred to a nitrocellulose membrane (GE Healthcare) using a semidry system (“Pegasus,” Gesellschaft für Phorese, Analytik und Separation GmbH). After blocking with 5% non-fat milk in TBST (10 mM Tris, pH 8.0, 150 mM NaCl, 0.5% Tween 20) for 60 min, membranes were incubated with primary antibodies against claudin 4 (1:200; Abcam), claudin 8 (1:250; Invitrogen), and REG3G (1:500; Elabscience Biotechnology Inc., TX, USA) overnight at 4°C using Roti^®^-Block buffer (Carl Roth GmbH + Co. KG, Karlsruhe, Germany). Membranes were washed four times for 10 min with TBST before and after 60 min incubation with the secondary antibody anti-rabbit antibody (1:2500, Abcam) at room temperature. Subsequently, membranes were incubated with a chemiluminescence solution (Clarity™ Western ECL Substrate, Bio-Rad) following manufacturer's instructions. After a stripping step with stripping buffer (1.5% glycine, 0.1% SDS, 1% Tween, pH 2.2) staining for GAPDH (1:10000; GenScript USA Inc., NJ, USA) followed on the same membrane. Blots were visualized using the ChemiDoc™ Touch Imaging System (Bio-Rad). Protein expression was evaluated by Image Lab™ Software (Bio-Rad). Results were shown as protein band intensity normalized to the GAPDH protein band.

### Statistical Analysis

All data were analyzed using GraphPad Prism 6^®^ software (GraphPad Software, La Jolla, USA). Parametric data were shown as mean ± SEM and non-parametric data as median ± interquartile range. All data were tested with the Shapiro-Wilk or Kolmogorov-Smirnov normality test for normal distribution. When the assumption about normally distributed data was not met, a non-parametric test was used. Statistical analysis for non-parametric data was performed using one-way ANOVA Kruskal-Wallis test with Dunn's multiple comparison test. For parametric data, one-way ANOVA with Sidak's test as *post-hoc* test was carried out. Statistical analysis of mucus layer thickness was performed using repeated measures one-way ANOVA Friedman test with Dunn's multiple comparison test. Comparison of data with two factors was analyzed with two-way ANOVA with Sidak's multiple comparisons test. *P* < 0.05 was considered significant (^*^P < 0.05, ^**^P < 0.01, ^***^P < 0.001, ^****^P < 0.0001).

## Results

### Minimal Bacterial Consortia, ASF, and OMM^12^, Stably Colonize GF *Il10*-deficient Mice

**Figure 1:**
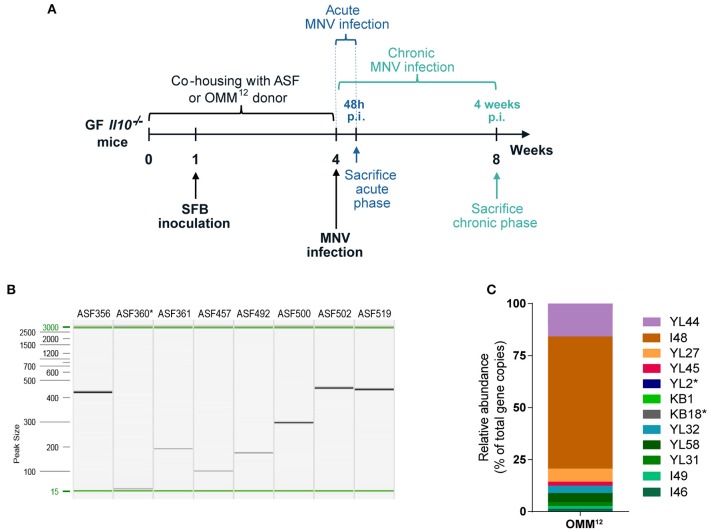
Gnotobiotic mouse model of experimental IBD. **(A)** Experimental design: GF *Il10*^−/−^ mice were colonized at weaning with ASF or OMM^12^ consortia by co-housing with minimal microbiota donor mice for 4 weeks. One week after co-housing commenced, mice were inoculated with SFB on 2 consecutive days. At the end of co-housing mice were infected with MNV for 4 weeks (chronic phase of MNV infection) or for 48 h (acute phase of MNV infection) and subsequently sacrificed. **(B)** Representative image of capillary gel electrophoresis showing ASF member-specific PCR products determined by strain-specific PCR assay in *Il10*^−/−^ mice after 4 weeks co-housing with ASF donor (*Clostridium sp*. ASF356, *Lactobacillus acidophilus* ASF360, *Lactobacillus murinus* ASF361, *Mucispirillum schaedleri* ASF457, *Eubacterium plexicaudatum* ASF492, *Pseudoflavonifractor sp*. ASF500, *Clostridium sp*. ASF502, and *Parabacteroides goldsteinii* ASF519); *n* = 15. **(C)** Representative OMM^12^ composition (relative abundance) determined by strain-specific qPCR assay in *Il10*^−/−^ mice after co-housing with OMM^12^ donor mouse for 4 weeks (*Akkermansia muciniphila* YL44, *Bacteroides caecimuris* I48, *Muribaculum intestinale* YL27, *Turicimonas muris* YL45, *Bifidobacterium longum* subsp. *animalis* YL2, *Enterococcus faecalis* KB1, *Acutalibacter muris* KB18, *Clostridium clostridioforme* YL32, *Blautia coccoides* YL58, *Flavonifractor plautii* YL31, *Lactobacillus reuteri* I49, and *Clostridium innocuum* I46); *n* = 16. GF, germ-free; ASF, Altered Schaedler Flora; OMM^12^, Oligo-Mouse-Microbiota 12; SFB, segmented filamentous bacteria; MNV, murine norovirus; p.i., post infection. ^*^Below detection limit.

GF *Il10*^−/−^ mice were colonized with minimal bacterial consortia, ASF or OMM^12^, by co-housing them with minimal microbiota donor mice for 4 weeks (Figure 1A). The microbiota transfer (co-housing) was initiated after weaning at the age of 4 weeks. Subsequently, these two minimal bacterial consortia were modulated by SFB and MNV infection. One week after co-housing began, mice were inoculated with SFB on 2 consecutive days. At the end of co-housing, 8 week old *Il10*^−/−^ mice were infected with MNV for 4 weeks (chronic MNV infection) or 48 h (acute MNV infection) and subsequently sacrificed (Figure 1A). At the age of 8 weeks, fecal samples were collected from gnotobiotic *Il10*^−/−^ mice and screened for the presence of minimal microbiota members. All ASF strains except ASF360 were detected in fecal samples by PCR analysis (Figure 1B). In *Il10*^−/−^ mice colonized with OMM^12^, the presence of 10 members was confirmed by qPCR, including YL44, I48, YL27, YL45, KB1, YL32, YL58, YL31, I49, and I46 (Figure 1C). OMM^12^ members YL2 and KB18 were not detected. However, all undetected bacteria (ASF360, YL2, and KB18) in gnotobiotic *Il10*^−/−^ mice were also not identified in minimal microbiota donor colonies, indicating that these bacteria are either absent or below the detection limit. Therefore, these results showed that members of both minimal consortia can be successfully transferred to *Il10*^−/−^ mice by co-housing.

### Severity of MNV-triggered Colitis Depends on the Presence of Specific Bacteria

**Figure 2:**
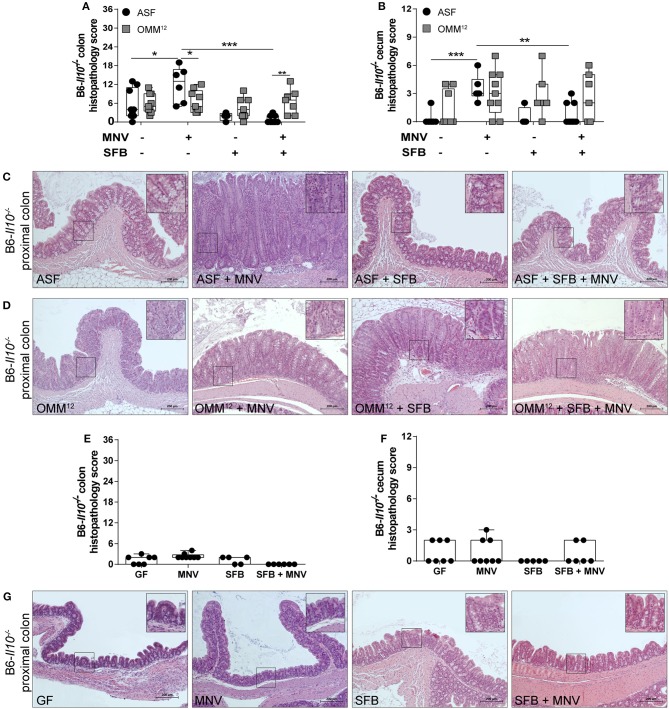
Histological analysis of intestinal tissues of gnotobiotic B6-*Il10*^−/−^ mice 4 weeks after MNV infection. **(A)** Histological score quantifying alterations observed in the colon tissue 4 weeks after MNV infection in ASF- or OMM^12^-colonized B6-*Il10*^−/−^ mice with or without SFB co-colonization. **(B)** Histological score quantifying alterations observed in the ceca 4 weeks after MNV infection in ASF- or OMM^12^-colonized B6-*Il10*^−/−^ mice with or without SFB co-colonization. Data presented in box and whiskers plots are the medians with minimum, maximum, and individual values obtained from two to three independent experiments (*n* = 5–9). **(C,D)** Representative images of H&E stained proximal colon sections of **(C)** ASF-colonized and **(D)** OMM^12^-colonized B6-*Il10*^−/−^ mice infected with MNV for 4 weeks and/or co-colonized with SFB. Scale bars: 200 μm. Insets: magnification of areas outlined by black boxes in main images. **(E)** Histological score quantifying alterations observed in the colon of GF B6-*Il10*^−/−^ mice 4 weeks after MNV infection with or without SFB monocolonization. **(F)** Histological score quantifying alterations observed in the ceca of GF B6-*Il10*^−/−^ mice 4 weeks after MNV infection with or without SFB monocolonization. Data presented in box and whiskers plots are the medians with minimum, maximum, and individual values obtained from one to two independent experiments (*n* = 5–7). **(G)** Representative images of H&E stained proximal colon tissue of GF B6-*Il10*^−/−^ mice, MNV-infected GF B6-*Il10*^−/−^ mice, SFB monocolonized B6-*Il10*^−/−^ mice and SFB monocolonized and MNV-infected B6-*Il10*^−/−^ mice. Scale bars: 200 μm. Insets: magnification of areas outlined by black boxes in main images. GF, germ-free; ASF, Altered Schaedler Flora; OMM^12^, Oligo-Mouse-Microbiota 12; SFB, segmented filamentous bacteria; MNV, murine norovirus; H&E, hematoxylin and eosin. Statistically significant diffrences are indicated as follows: ^*^P < 0.05, ^**^P < 0.01 and ^***^P < 0.001.

*Il10*^−/−^ mice develop spontaneous colitis, which is strongly dependent on the microbiota (19). Additionally, MNV has been shown to trigger colitis in these mice in a microbiota-dependent manner (22). In our study, we first assessed the impact of minimal bacterial consortia alone on colitis development in *Il10*^−/−^ mice. ASF and OMM^12^ induced none to mild histological changes in the colon and cecum of 12 week old B6-*Il10*^−/−^ mice (Figures 2A–D). These changes were mainly characterized by infiltration of myeloid cells such as lymphocytes in the lamina propria. MNV infection exacerbated the severity of colitis only in B6-*Il10*^−/−^ mice that were colonized with ASF, causing moderate intestinal inflammation (Figures 2A–C). In contrast, B6-*Il10*^−/−^ mice colonized with OMM^12^ were unaffected by MNV infection (Figures 2A,B,D). Pathological lesions in ASF-associated B6-*Il10*^−/−^ mice were predominantly located in the proximal colon and characterized by hyperplasia of the crypt epithelium and infiltration of inflammatory cells such as lymphocytes and granulocytes to the tela submucosa and tunica muscularis (Figure 2C). The middle and distal part of the colon were less affected.

**Figure 3:**
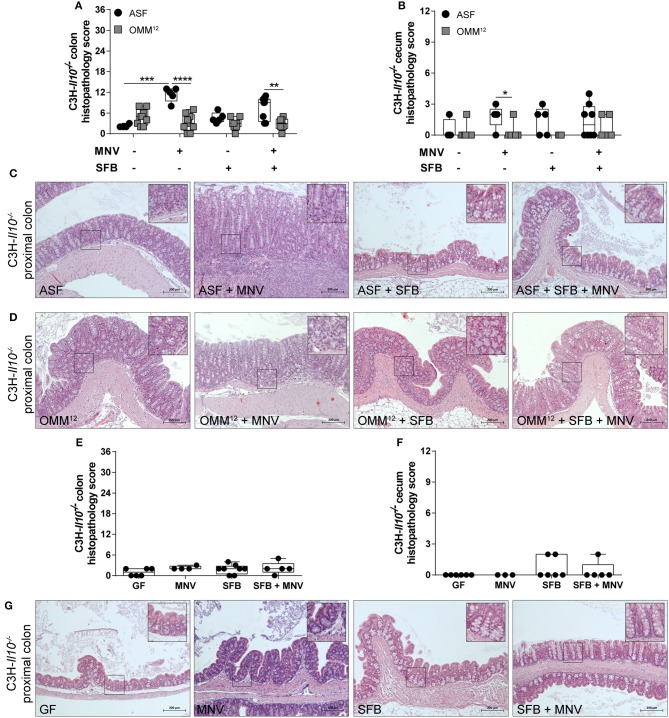
Histological analysis of intestinal tissues of gnotobiotic C3H-*Il10*^−/−^ mice 4 weeks after MNV infection. **(A)** Histological score quantifying alterations observed in the colon tissue 4 weeks after MNV infection in ASF- or OMM^12^-colonized C3H-*Il10*^−/−^ mice with or without SFB co-colonization. **(B)** Histological score quantifying alterations observed in the ceca 4 weeks after MNV infection in ASF- or OMM^12^-colonized C3H-*Il10*^−/−^ mice with or without SFB co-colonization. Data presented in box and whiskers plots are the medians with minimum, maximum, and individual values obtained from one to three independent experiments (*n* = 4–9). **(C,D)** Representative images of H&E stained proximal colon sections of **(C)** ASF-colonized and **(D)** OMM^12^-colonized C3H-*Il10*^−/−^ mice infected with MNV for 4 weeks, and/or co-colonized with SFB. Scale bars: 200 μm. Insets: magnification of areas outlined by black boxes in main images. **(E)** Histological score quantifying alterations observed in the colon of GF C3H-*Il10*^−/−^ mice 4 weeks after MNV infection with or without SFB monocolonization. **(F)** Histological score quantifying alterations observed in the ceca of GF C3H-*Il10*^−/−^ mice 4 weeks after MNV infection with or without SFB monocolonization. Data presented in box and whiskers plots are medians with minimum, maximum, and individual values obtained from one to three independent experiments (*n* = 4–8). **(G)** Representative images of H&E stained proximal colon tissue of GF C3H-*Il10*^−/−^ mice, MNV-infected GF C3H-*Il10*^−/−^ mice, SFB monocolonized C3H-*Il10*^−/−^ mice and SFB monocolonized and MNV-infected C3H-*Il10*^−/−^ mice. Scale bars: 200 μm. Insets: magnification of areas outlined by black boxes in main images. GF, germ-free; ASF, Altered Schaedler Flora; OMM^12^, Oligo-Mouse-Microbiota 12; SFB, segmented filamentous bacteria; MNV, murine norovirus; H&E, hematoxylin and eosin. Statistically significant differences are indicated as follows: ^*^P < 0.05, ^**^P < 0.01, ^***^P < 0.001 and ^****^P < 0.0001.

To investigate whether the impact of minimal consortia on the development of MNV-triggered colitis depends on the host genetic background, C3H-*Il10*^−/−^ mice were colonized with ASF or OMM^12^ consortium and subsequently infected with MNV. Uninfected ASF-colonized C3H-*Il10*^−/−^ mice showed no histopathological changes in either the colon or cecum (Figures 3A–C). In contrast, uninfected OMM^12^-associated C3H-*Il10*^−/−^ mice showed mild histopathological changes mainly in the colon characterized by infiltration of lymphocytes (Figures 3A,B,D). MNV infection triggered colitis only in C3H-*Il10*^−/−^ mice colonized with ASF, but not in those carrying OMM^12^ (Figures 3A–D). The histopathological scores between ASF-colonized B6-*Il10*^−/−^ and C3H-*Il10*^−/−^ mice were comparable with or without MNV infection, indicating that under these conditions both strains show similar colitis susceptibility. The same results were also observed between OMM^12^-colonized B6-*Il10*^−/−^ and C3H-*Il10*^−/−^ mice. In addition, and in line with our previous report, MNV alone did not cause pathological changes in the gut of GF B6-*Il10*^−/−^ or C3H-*Il10*^−/−^ mice (Figures 2E–G, 3E–G). These results indicated that the ability of MNV to trigger colitis depends on the presence of specific bacteria.

### SFB Co-colonization Abolishes MNV-induced Colitis Only in Mice Colonized With ASF

Subsequently, we modulated ASF and OMM^12^ consortia by co-colonizing *Il10*^−/−^ mice with SFB at the age of 5 weeks. These commensal bacteria show potent immunostimulatory effects, but are also discussed to be pathobionts (32). SFB co-colonization did not exacerbate the histopathology score in the colon or cecum of 12 week old B6-*Il10*^−/−^ mice colonized with ASF or OMM^12^ consortium. Moreover, when ASF-associated B6-*Il10*^−/−^ mice were infected with MNV, the presence of SFB abolished MNV-triggered colitis in these mice. The histopathology score was significantly reduced in both the colon and cecum of B6-*Il10*^−/−^ mice (Figures 2A,B). Furthermore, microscopic analysis of H&E stained colon tissue sections showed restoration of normal intestinal morphology (Figure 2C). In contrast, histological changes in the gut of OMM^12^-associated B6-*Il10*^−/−^ mice were not markedly modulated by either MNV or SFB (Figures 2A,B,D). Similar results were also observed in C3H-*Il10*^−/−^ mice, however, the SFB-mediated protective effect was less pronounced in these mice (Figures 3A–D). Furthermore, SFB monocolonization as well as SFB and MNV co-infection did not cause pathological changes in the gut epithelium of both analyzed *Il10*^−/−^ mouse strains (Figures 2E–G, 3E–G). Together, these results suggested that SFB co-colonization protects against pathological changes.

### Host Response Is Distinctively Modulated by Different Microbiota Compositions

**Figure 4:**
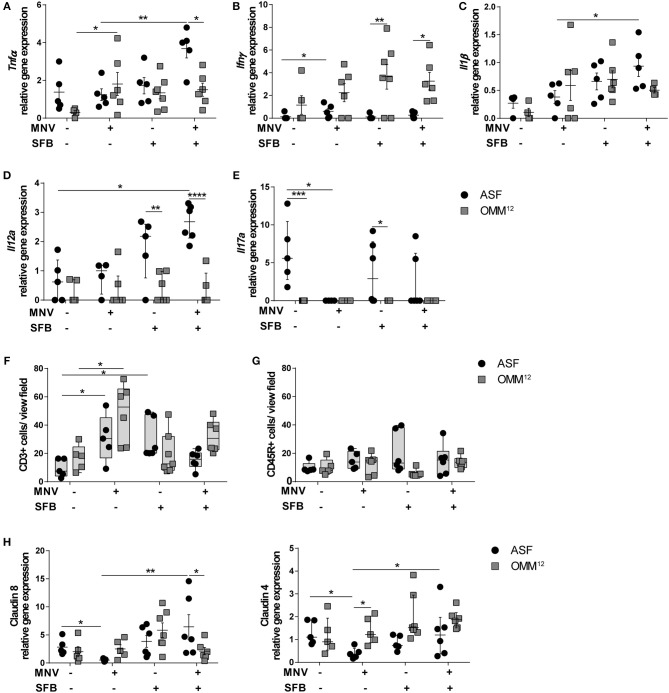
Different intestinal microbiota compositions distinctively modulate host response. **(A–E)** Gene expression of pro-inflammatory cytokines **(A)** *Tnf*α, **(B)** *Ifn*γ, **(C)** *Il1*β, **(D)** *Il12a*, and **(E)** *Il17a* measured by qPCR in total RNA isolated from proximal colon of ASF-colonized or OMM^12^-colonized B6-*Il10*^−/−^ mice 4 weeks after MNV infection with or without SFB co-colonization. Relative differences in gene expression were calculated by the comparative 2^−ΔCt^ method. Parametric data were shown as mean ± SEM (*n* = 5–7). Non-parametric data were shown as median ± interquartile range (*n* = 5–7). Data were obtained from one to two independent experiments. **(F,G)** Quantification of **(F)** CD3+ and **(G)** CD45R+ cells in the colon tissue of ASF- or OMM^12^-colonized B6-*Il10*^−/−^ mice with or without SFB co-colonization 4 weeks after MNV infection. Data presented in box and whiskers plots are medians with minimum, maximum, and individual values obtained from one to two independent experiments (*n* = 5–7). **(H)** Gene expression of tight junction genes claudin 8 and claudin 4 measured by qPCR in total RNA isolated from proximal colon of ASF- or OMM^12^-colonized B6-*Il10*^−/−^ mice with or without SFB co-colonization 4 weeks after MNV infection. Parametric data were shown as mean ± SEM (*n* = 5–7). Non-parametric data were shown as median ± interquartile range (*n* = 5–7). Data were obtained from one to two independent experiments. ASF, Altered Schaedler Flora; OMM^12^, Oligo-Mouse-Microbiota 12; SFB, segmented filamentous bacteria; MNV, murine norovirus; *Tnf*α, Tumor necrosis factor alpha; *Ifn*γ, Interferon gamma; *Il1*β, Interleukin 1 beta; *Il12a*, Interleukin 12a; *Il17a*, Interleukin 17a. Statistically significant differences are indicated as follows: ^*^P < 0.05, ^**^P < 0.01, ^***^P < 0.001 and ^****^P < 0.0001.

Next, we determined the expression of pro-inflammatory cytokines in the proximal colon to investigate the signaling molecules that are produced by the host upon encountering specific microorganisms. B6-*Il10*^−/−^ mice harboring different minimal bacterial consortia expressed partially distinct cytokines (Figures 4A–E). Twelve week old ASF-associated B6-*Il10*^−/−^ mice showed higher expression of *Tnf*α and lower expression of *Ifn*γ than OMM^12^-colonized B6-*Il10*^−/−^ mice (Figures 4A,B). In mice colonized either with ASF or OMM^12^, gene expression of *Il1*β and *Il12a* was similar (Figures 4C,D). Four weeks after MNV infection, OMM^12^-associated mice produced more *Tnf*α than ASF-associated mice in which no upregulation of this cytokine was observed upon infection (Figure 4A). However, ASF-colonized mice showed slight increase of *Ifn*γ expression upon infection (Figure 4B). Co-colonization of ASF-associated mice with SFB induced higher expression of pro-inflammatory cytokines such as *Tnf*α, *Il1*β, and *Il12a* upon MNV infection compared to infected ASF-associated mice without SFB (Figures 4A,C,D). The expression of these cytokines was not elevated in infected OMM^12^-associated mice co-colonized with SFB. Expression of *Ifn*γ was slightly reduced in uninfected and infected ASF-colonized mice upon SFB co-colonization, but remained unchanged in mice associated with OMM^12^ (Figure 4B). *Il17a* was expressed in ASF-colonized B6-*Il10*^−/−^ mice co-colonized with SFB with and without MNV infection, but also in mice only carrying ASF. In contrast, no *Il17a* expression was detected in all groups of OMM^12^-associated B6-*Il10*^−/−^ mice. Upon MNV infection, *Il17a* expression was abolished in ASF-colonized B6-*Il10*^−/−^ mice without SFB (Figure 4E).

To determine the cells present in the gut epithelium of these mice, colon tissue sections were stained immunohistochemically for the presence of myeloid CD3+ (predominantly T cells) and CD45R+ cells (naïve B cells). MNV infection elevated the number of CD3+ cells in the lamina propria of mice colonized with either minimal consortium (Figure 4F). SFB co-colonization increased the number of CD3+ cells in ASF-colonized B6-*Il10*^−/−^ mice, but not in OMM^12^-associated B6-*Il10*^−/−^ mice. Furthermore, when comparing MNV-infected groups only, SFB colonization reduced the number of CD3+ cells in ASF- and OMM^12^-colonized mice 4 weeks after MNV infection. However, this effect was more pronounced in ASF-colonized mice (Figure 4F). Moreover, staining for CD45R+ cells showed no difference among all groups (Figure 4G).

As MNV-triggered inflammation is associated with barrier disruption, we analyzed whether these two minimal consortia differently affect MNV-induced barrier disruption by downregulating gene expression of tight junction (TJ) components. Therefore, we measured the gene expression of claudin 4 and 8, whose downregulation is associated with increased intestinal permeability (22, 40, 41). MNV infection significantly reduced gene expression of claudin 4 and 8 in the proximal colon of 12 week old ASF-associated B6-*Il10*^−/−^ mice. MNV was not able to downregulate the gene expression of these claudins in mice colonized with OMM^12^. Furthermore, when ASF-associated mice were co-colonized with SFB, MNV infection did not downregulate the gene expression of these TJ proteins (Figure 4H). Co-colonization with SFB did not significantly change the expression of claudin 4 and 8 in mice carrying OMM^12^ (Figure 4H). This indicates that the protective effect provided by SFB is partially mediated by strengthening the epithelial barrier.

### SFB Co-colonization Depends on the Microbiota Composition

**Figure 5:**
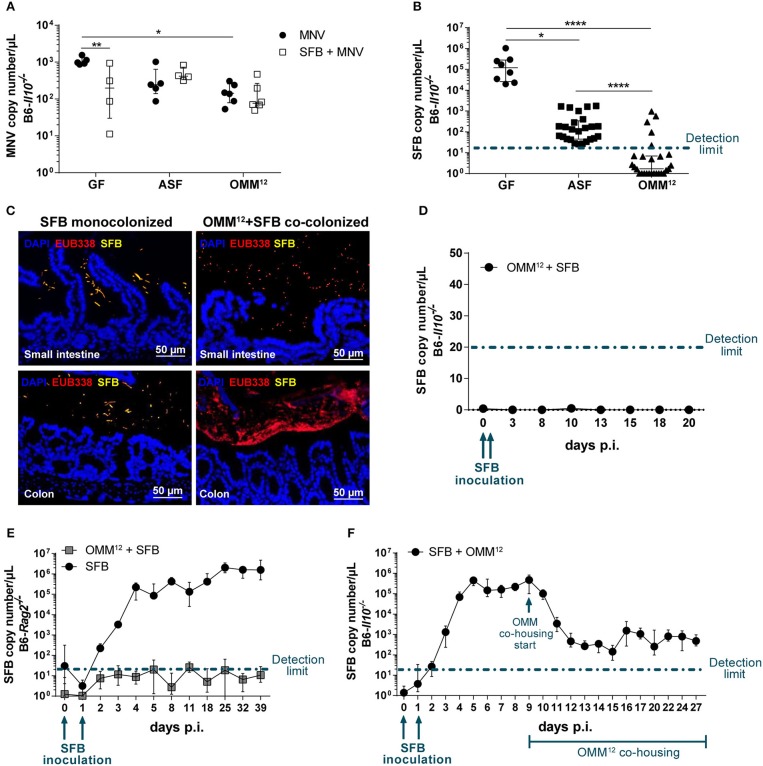
SFB co-colonization depends on the microbiota composition. **(A)** Using virus-specific qPCR, viral load was measured in total RNA isolated from the proximal colon of GF, ASF- and OMM^12^-colonized B6-*Il10*^−/−^ mice infected with MNV for 4 weeks with or without co-colonization with SFB. Data were shown as median ± interquartile range (*n* = 4–6). Data were collected from one to two independent experiments. **(B)** SFB copy numbers were measured in total DNA isolated from feces of 12 week old GF, ASF-, or OMM^12^-associated B6-*Il10*^−/−^ mice co-colonized with SFB with or without MNV infection for 4 weeks. Data were shown as median ± interquartile range and collected from three to nine independent experiments (*n* = 8–27). **(C)** Representative images of FISH staining of ileal and proximal colon tissue sections of GF and OMM^12^-associated B6-*Il10*^−/−^ mice co-colonized with SFB (*n* = 5). DNA was stained using DAPI (blue), SFB were detected using an SFB-specific probe (yellow) and all bacteria were detected by the EUB338 probe (red). Scale bars: 50 μm. **(D)** SFB colonization kinetic was determined by using an SFB-specific qPCR measuring SFB copy numbers in the total DNA isolated from feces of OMM^12^-colonized B6-*Il10*^−/−^ mice over a period of 20 days. OMM^12^-colonized B6-*Il10*^−/−^ mice were inoculated with the gut content of SFB monocolonized mice at day 0 and 1. Data were shown as median ± interquartile range (*n* = 7). Data were obtained from two independent experiments. **(E)** SFB colonization kinetics were determined by an SFB-specific qPCR measuring SFB copy numbers in the total DNA isolated from feces of OMM^12^-colonized B6-*Rag2*^−/−^ mice over a period of 39 days. OMM^12^-colonized B6-*Rag2*^−/−^ mice were inoculated with the gut content of SFB monocolonized mice at day 0 and 1. Data were shown as median ± interquartile range (*n* = 7). Data were obtained from two independent experiments. **(F)** SFB colonization kinetics were determined by an SFB-specific qPCR measuring SFB copy numbers in the total DNA isolated from feces of B6-*Il10*^−/−^ mice first monocolonized with SFB and subsequently colonized with OMM^12^. GF B6-*Il10*^−/−^ mice were inoculated with the gut content of SFB monocolonized mice at day 0 and 1. OMM^12^ consortium transfer started when stable SFB colonization was established. Data were shown as median ± interquartile range (*n* = 7). Data were obtained from two independent experiments. GF, germ-free; ASF, Altered Schaedler Flora; OMM^12^, Oligo-Mouse-Microbiota 12; SFB, segmented filamentous bacteria; MNV, murine norovirus; FISH, fluorescent *in situ* hybridization. Dash-dotted line: detection limit. Statistically significant differences are indicated as follows: ^*^P < 0.05, ^**^P < 0.01 and ^****^P < 0.0001.

To exclude that differences in the MNV infection rate are responsible for observed differences in intestinal pathology, we measured the viral load in total RNA isolated from the proximal colon using virus-specific qPCR. MNV copy numbers decreased when mice were colonized with bacteria (Figure 5A). However, no correlation between the MNV load and intestinal pathology was found, as the highest viral load was detected in GF B6-*Il10*^−/−^ mice that showed no intestinal pathology (Figures 2E,G, 5A). Additionally, SFB inoculation did not significantly decrease the viral load in mice carrying minimal consortia suggesting that the SFB-mediated protective effect is not facilitated by preventing MNV infection (Figure 5A).

As the SFB-mediated protective effect was absent in B6-*Il10*^−/−^ mice colonized with OMM^12^, we investigated the SFB colonization rate. Using an SFB-specific qPCR assay we quantified SFB in feces of GF, ASF-, and OMM^12^-colonized B6-*Il10*^−/−^ mice 7 weeks after SFB co-colonization. The presence of SFB was confirmed only in GF and ASF-colonized mice ranging from ~10^2^ to 10^3^ copies/μL in ASF-colonized mice and 10^5^ copies/μL in SFB monocolonized mice. In contrast, SFB remained largely undetected in mice carrying OMM^12^ (Figure 5B). To confirm the absence of SFB in OMM^12^-colonized mice, we performed fluorescence *in situ* hybridization (FISH) by staining ileum and colon sections with SFB-specific and bacteria domain-specific (EUB338) probes. SFB were detected and localized near the intestinal epithelium mainly in the ileum, but also in the colon of SFB monocolonized mice. SFB were not detected in OMM^12^-associated mice (Figure 5C). As we could not detect SFB 7 weeks post inoculation, we questioned whether SFB could colonize OMM^12^-associated mice at all or whether they were eradicated over the course of the experiment. To test this, we followed SFB colonization kinetics in OMM^12^-associated mice over 20 days post SFB inoculation. OMM^12^-associated B6-*Il10*^−/−^ mice were inoculated with the gut content of SFB monocolonized mice at day 0 and 1. Feces samples were collected every few days and SFB-specific qPCR was used to determine the presence of SFB. Over the course of 20 days, SFB were below the detection limit, demonstrating that under this experimental condition SFB are unable to colonize OMM^12^-associated mice (Figure 5D). These results indicated that the absence of the SFB-mediated protective effect in mice colonized with OMM^12^ is likely due to lack of SFB colonization. Furthermore, to analyze whether the adaptive immunity in OMM^12^-associated mice plays a role in SFB colonization, we colonized GF B6-*Rag2*^−/−^ mice with the OMM^12^ consortium and SFB, and again followed SFB colonization kinetics. SFB successfully colonized GF B6-*Rag2*^−/−^ mice. However, SFB could not colonize OMM^12^-associated B6-*Rag2*^−/−^ mice indicating that the adaptive immune response is not responsible for preventing SFB colonization (Figure 5E). Additionally, to analyze whether the absence of SFB is due to colonization resistance provided by OMM^12^ consortium, we reversed the order of colonization. Juvenile GF B6-*Il10*^−/−^ mice were first inoculated with SFB and co-housing with an OMM^12^ donor was initiated when GF B6-*Il10*^−/−^ mice were stably monocolonized with SFB. The presence of SFB was detected in feces of monocolonized B6-*Il10*^−/−^ mice starting at day 3 post inoculation (p.i.) and reached its maximum at day 5 p.i. At day 9 p.i., SFB monocolonized mice were co-housed with an OMM^12^ donor. The SFB load exponentially decreased but stabilized at 10^2^-10^3^ copies/μL from day 12 p.i., indicating that SFB can compete with OMM^12^ members only when they colonize first (Figure 5F). In addition, SFB copy numbers detected upon colonization with OMM^12^ were comparable with those that were observed in ASF-SFB co-colonized mice (Figure 5B).

### SFB Co-colonization of ASF-associated B6-*Il10^−/−^* Mice Boosted Epithelial Barrier Defense and Immune Response in the Chronic and Acute Phase of MNV Infection

**Figure 6:**
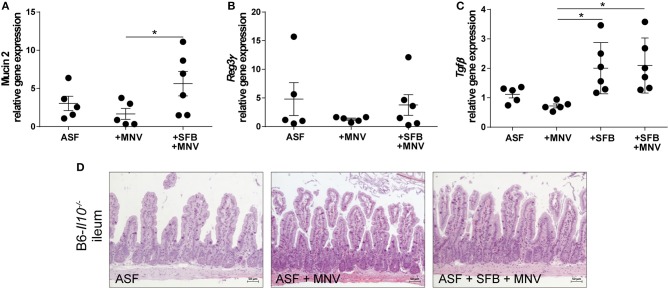
The presence of SFB enhances expression of intestinal barrier defense and immune regulatory factors in the chronic phase of MNV infection. **(A–C)** Gene expression of **(A)** *Muc2*, **(B)** *Reg3*γ, and **(C)** *Tgf*β measured by qPCR in total RNA isolated from the proximal colon of ASF-colonized B6-*Il10*^−/−^ mice infected with MNV for 4 weeks with or without SFB co-colonization. Relative differences in gene expression were calculated by the comparative 2^−ΔCt^ method. Parametric data were shown as mean ± SEM (*n* = 5–6). Data were obtained from one to two independent experiments. **(D)** Representative images of H&E stained ileum tissue sections of ASF-associated B6-*Il10*^−/−^ mice infected with MNV for 4 weeks with and without SFB co-colonization (*n* = 5). ASF, Altered Schaedler Flora; SFB, segmented filamentous bacteria; MNV, murine norovirus; H&E, hematoxylin and eosin; h, hours; *Muc2*, Mucin 2; *Reg3*γ, Regenerating islet-derived 3 gamma; *Tgf*β, Transforming growth factor beta.1. Statistically significant differences are indicated as follows: ^*^P < 0.05.

Next, we were interested in elucidating SFB-mediated protective effects that prevented MNV to induce intestinal inflammation in B6-*Il10*^−/−^ mice associated with ASF. As we already mentioned previously, SFB promoted barrier integrity by preventing downregulation of tight junction components claudin 4 and 8 in the chronic phase of MNV infection (4 weeks post infection) (Figure 4H). However, the presence of SFB was also associated with increased expression of pro-inflammatory cytokines such as *Tnf*α, *Il1*β, *Il12a*, and *Il17a* even though no inflammatory lesions were observed in the gut of these animals (Figures 2A,C, 4A). Therefore, we analyzed expression of other barrier-determining factors such as mucus 2 and an antimicrobial peptide produced by epithelial cells, *Reg3*γ. Furthermore, we also measured expression of the regulatory cytokine *Tgf*β. These analyses showed that in the chronic phase of MNV infection in ASF-colonized mice, the presence of SFB increased production of *Muc2*, a major constituent of the mucus layer (Figure 6A). The expression of *Reg3*γwas slightly increased in ASF mice co-colonized with SFB 4 weeks after MNV infection (Figure 6B). Furthermore, the presence of SFB also significantly increased the expression of *Tgf*β in uninfected and chronically MNV-infected ASF-associated mice (Figure 6C). These results strengthened the hypothesis that the protective effects of SFB are mediated by enhancing intestinal barrier defense and host immune response.

As the ileum is the predominant SFB habitat, we investigated whether SFB or MNV cause pathological changes in the small intestine by staining ileal tissue with H&E. ASF-colonized and ASF-SFB co-colonized mice did not show any signs of ileitis or changes in ileal histomorphology 4 weeks after MNV infection (Figure 6D).

**Figure 7:**
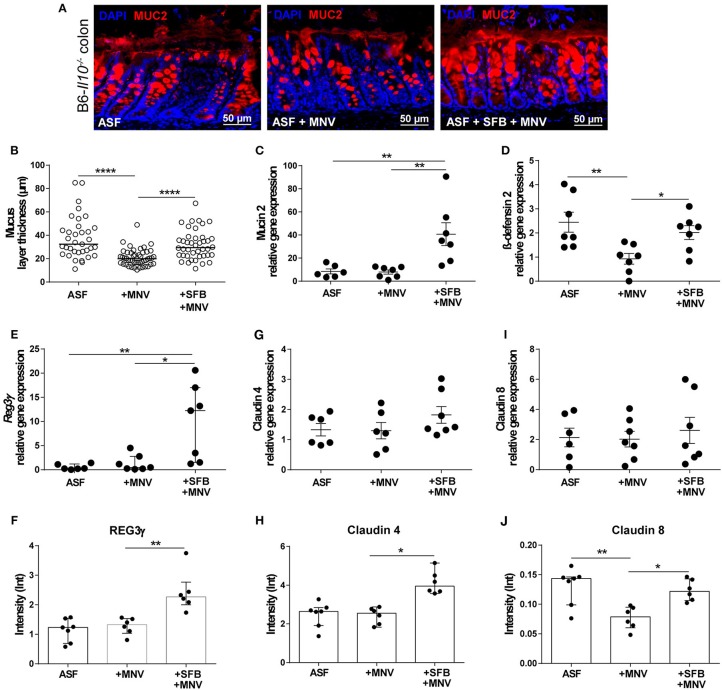
SFB-mediated protective effect in the acute phase of MNV infection enhances intestinal epithelial barrier defense mechanisms. **(A)** Representative images of immunofluorescence staining of MUC2 (red) on colon sections obtained from ASF-associated B6-*Il10*^−/−^ mice infected with MNV for 48 h with or without SFB co-colonization. Nuclei (blue) were counterstained with DAPI (*n* = 7). Scale bars: 50 μm. **(B)** Quantification of MUC2 immunofluorescent stained layer thickness measured in the colon of ASF-associated B6-*Il10*^−/−^ mice 48 h after MNV infection with or without SFB so-colonization. Colon tissue sections of seven mice per group were blindly analyzed. Six images per tissue section were taken and five measurements of layer thickness per image were recorded using Zeiss ZEN blue software. Subsequently one mean value of layer thickness per image was generated. In total six technical replicates per animal are shown. Bars represent the median. Data were obtained from one to two independent experiments. **(C)** Gene expression of *Muc2* measured by qPCR in total RNA isolated from the proximal colon of ASF-colonized B6-*Il10*^−/−^ mice 48 h after MNV infection with and without SFB co-colonization. Relative differences in gene expression were calculated by the comparative 2^−ΔCt^ method (*n* = 6–7). **(D,E)** Gene expression of antimicrobial peptides **(D)** β-defensin 2 and **(E)** *Reg3*γ measured by qPCR in total RNA isolated from proximal colon of ASF-colonized B6-*Il10*^−/−^ mice 48 h after MNV infection with and without SFB co-colonization. Relative differences in gene expression were calculated by the comparative 2^−ΔCt^ method (*n* = 6–7). **(F)** Densitometric quantification of REG3γ protein expression measured in colon lysates of ASF-colonized B6-*Il10*^−/−^ mice 48 h post MNV infection with and without SFB co-colonization normalized to GAPDH (*n* = 6–7). Blot images were analyzed by Image Lab^TM^ Software. **(G)** Gene expression of claudin 4 measured by qPCR in total RNA isolated from proximal colon of ASF-colonized B6-*Il10*^−/−^ mice 48 h after MNV infection with and without SFB co-colonization. Relative differences in gene expression were calculated by the comparative 2^−ΔCt^ method (*n* = 6–7). **(H)** Densitometric quantification of claudin 4 protein expression measured in colon lysates of ASF-colonized B6-*Il10*^−/−^ mice 48 h post MNV infection with and without SFB co-colonization normalized to GAPDH (*n* = 6–7). Blot images were analyzed by Image Lab^TM^ Software. **(I)** Gene expression of claudin 8 measured by qPCR in total RNA isolated from proximal colon of ASF-colonized B6-*Il10*^−/−^ mice 48 h post MNV infection with and without SFB co-colonization. Relative differences in gene expression were calculated by the comparative 2^−ΔCt^ method (*n* = 6–7). **(J)** Densitometric quantification of claudin 8 protein expression measured in colon lysates of ASF-colonized B6-*Il10*^−/−^ mice 48 h after MNV infection with and without SFB co-colonization normalized to GAPDH (*n* = 6–7). Blot images were analyzed by Image Lab^TM^ Software. Parametric data were presented as mean ± SEM. Non-parametric data were presented as median ± interquartile range. Data were obtained from one to two independent experiments. GF, germ-free; ASF, Altered Schaedler Flora; SFB, segmented filamentous bacteria; MNV, murine norovirus; h, hours; MUC2, Mucin 2; REG3γ, Regenerating islet-derived 3 gamma. Statistically significant differences are indicated as follows: ^*^P < 0.05, ^**^P < 0.01 and ^****^P < 0.0001.

In our previous study, we showed that MNV infection at an early stage (48 h) post infection induces structural and functional intestinal barrier changes that are hypothesized to be the initiating factor in the development of observed intestinal inflammation. Therefore, we analyzed whether the presence of SFB prevents MNV-induced barrier damage in the acute phase of MNV infection. Thus, ASF-SFB co-colonized B6-*Il10*^−/−^ mice were sacrificed 48 h post MNV infection and expression of barrier-determining factors as well as the host immune response were analyzed. Acute MNV infection resulted in reduced thickness of the mucus layer in the colon of ASF-colonized mice, which was prevented when these mice were co-colonized with SFB (Figures 7A,B). Furthermore, the presence of SFB enhanced mucin 2 gene expression and MUC2 abundance in the colon of ASF-colonized mice (Figures 7A–C). Additionally, MNV infection reduced the gene expression of ß-defensin 2, an antimicrobial peptide, in ASF-colonized mice. However, when SFB were present, MNV did not downregulate ß-defensin 2 expression (Figure 7D). SFB co-colonization increased *Reg3*γ gene expression and protein production (Figures 7E,F). Even though no changes in the gene expression of claudin 4 and 8 were observed, at the protein level MNV infection reduced the expression of claudin 8 (Figures 7G,I,J). Furthermore, the expression of tight junction proteins, claudin 4 and 8, was upregulated 48 h after MNV infection only when SFB were present (Figures 7H,J).

**Figure 8:**
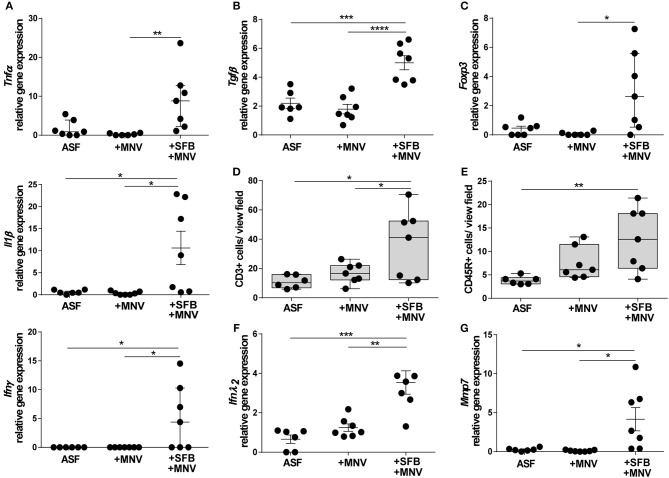
SFB-mediated protective effect in the acute phase of MNV infection enhances host immune responses. **(A–C)** Gene expression of **(A)** pro-inflammatory cytokines *Tnf*α, *Il1*β, and *Ifn*γ, **(B)** regulatory cytokine *Tgf*β, and **(C)** *Foxp3* measured by qPCR in total RNA isolated from proximal colon of ASF-colonized B6-*Il10*^−/−^ mice 48 h after MNV infection with and without SFB co-colonization. Relative differences in gene expression were calculated by the comparative 2^−ΔCt^ method (*n* = 7). **(D,E)** Quantification of **(D)** CD3+ and **(E)** CD45R+ cells in the colon tissue of ASF-colonized B6-*Il10*^−/−^ mice 48 h post MNV infection with and without SFB co-colonization. Data presented in box and whiskers plots are medians with minimum, maximum, and individual values (*n* = 7). **(F,G)** Gene expression of **(F)** *Ifn*λ_2_ and **(G)** *Mmp7* measured by qPCR in total RNA isolated from proximal colon of ASF-colonized B6-*Il10*^−/−^ mice 48 h post MNV infection with and without SFB co-colonization. Relative differences in gene expression were calculated by the comparative 2^−ΔCt^ method (*n* = 6–7). Parametric data were presented as mean ± SEM and non-parametric data as median ± interquartile range. Data were obtained from one to two independent experiments. GF, germ-free; ASF, Altered Schaedler Flora; SFB, segmented filamentous bacteria; MNV, murine norovirus; h, hours; *Tnf*α, Tumor necrosis factor alpha; *Il1*β, Interleukin 1 beta; *Ifn*γ, Interferon gamma; *Tgf*β, Transforming growth factor beta; *Foxp 3*, Forkhead box P3*; Ifn*λ_2_, Interferon lambda 2; *Mmp7*, Matrix metallopeptidase 7. Statistically significant differences are indicated as follows: ^*^P < 0.05, ^**^P < 0.01, ^***^P < 0.001 and ^****^P < 0.0001.

The impact of SFB co-colonization on the host immune system in the acute phase of MNV infection was also investigated. In ASF-SFB co-colonized mice strongly activated immune response was detected. SFB enhanced expression of pro-inflammatory cytokines such as *Tnf*α, *Il1*β, and *Ifn*γ in the colon (Figure 8A). However, the presence of SFB markedly upregulated expression of the regulatory cytokine *Tnf*β, but also enhanced the expression of *Foxp3* that encodes for a transcriptional regulator protein involved in the development and function of regulatory T cells (Figures 8B,C). Surprisingly, 48 h post MNV infection elevated levels of measured pro-inflammatory cytokines were not observed. Furthermore, the presence of SFB also increased the number of CD3+ and CD45R+ cells in the colon lamina propria (Figures 8D,E). As interferon type III is important for epithelial defense against viruses, the expression of *Ifn*λ_2_ in the colon was measured 48 h post MNV infection. The presence of SFB strongly enhanced expression of *Ifn*λ_2_, whereas MNV infection alone did not (Figure 8F). In addition, expression of *Mmp7*, a gene that is predominantly induced by *Ifn*λ_2_, was strongly upregulated in ASF mice that were co-colonized with SFB (Figure 8G). Altogether, our results showed that SFB-mediated protective effects in both chronic and acute phase of MNV infection were linked to improved intestinal epithelial barrier defense and also enhanced host immune response.

## Discussion

Mucosal surfaces are colonized with a complex community of microorganisms that play an important role in host health and disease (5). However, underlying mechanisms of how the intestinal microbiota and its particular members make or break intestinal homeostasis are still mainly unknown. Thus, gnotobiotic models evolved as powerful tools to mechanistically analyze host-microbiome interactions under standardized conditions. In this study, we employed a gnotobiotic model of experimental IBD and demonstrated that the outcome of virus-induced colitis in *Il10*^−/−^ mice depends on the composition of the intestinal microbiota.

Murine noroviruses (MNVs) are single-stranded RNA viruses that are highly prevalent in mouse colonies (42). This group of viruses is related to human noroviruses that are the main cause of non-bacterial gastroenteritis and are implicated in disease severity of IBD patients (43–45). Furthermore, MNVs were shown to induce and exacerbate intestinal inflammation in experimental models of IBD such as *Il10*^−/−^, *Atg16L1*^−/−^, and *Mdr1a*^−/−^ mice (22, 46, 47). MNV-induced intestinal pathology is microbiota-dependent, as GF or antibiotic treated mice do not develop inflammation after MNV infection (22, 48, 49). However, it is still unknown how different microbial communities help to facilitate norovirus infection. Here, we showed that the severity of MNV-triggered colitis in B6-*Il10*^−/−^ and C3H-*Il10*^−/−^ mice depends on the presence of specific bacteria. MNV exacerbated colitis and induced moderate intestinal inflammation in *Il10*^−/−^ mice colonized with ASF, a minimal microbiota composed of eight bacterial species. However, the histopathology score in mice associated with OMM^12^, a minimal bacterial consortium of 12 bacterial species, was not affected by MNV infection. Inflammatory lesions in *Il10*^−/−^ mice normally first appear in the cecum and proximal colon and involve the lower colon and rectum as disease progresses (50). Interestingly, Kuhn et al. showed that the localization of inflammatory lesions in *Il10*^−/−^ mice can be affected by the intestinal microbial composition. They described that *Il10*^−/−^ mice housed in a specific pathogen free environment develop an attenuated disease with lesions restricted to the proximal colon, while *Il10*^−/−^ mice housed under conventional conditions develop enterocolitis affecting the entire large intestine (15). In our model, MNV-induced inflammatory lesions in ASF-colonized mice predominately localized to the cecum and proximal colon, whereas the middle and distal colon were less affected. Therefore, a defined microbial environment represents a low colitogenic stimulus as defined by the degree and extent of inflammation.

SFB are commensals of a specific morphology that were first identified in the gut of rodents, but subsequently, morphologically similar species were detected in other organisms (32, 51–53). These bacteria live attached to intestinal epithelial cells and potently stimulate immune responses, especially the Th17 response (54). SFB have been shown to exert both protective effects in infection models and adverse effects in inflammation models (31, 55–57). In our study, we modified ASF and OMM^12^ consortia with SFB. SFB co-colonization abolished signs of MNV-triggered inflammation in ASF-colonized mice demonstrating SFB-mediated protective effects on colitis development. In contrast, histopathological scores of OMM^12^-colonized mice were not affected by SFB colonization.

Colonization processes and infections modulate host immunity. MNV infection activates interferon (IFN) and TNFα immune response pathways (58). Moreover, interferon type I and II responses are important in limiting MNV pathogenesis (59–61). In contrast, colitis development in *Il10*^−/−^ mice is driven by an aberrant response of Th1 cells and exaggerated production of pro-inflammatory cytokines such as IFNγ, IL17, and IL12 to microbiota-derived antigens (18). In our study, immunohistological staining of the colon showed increased infiltration of lymphocytes that were mainly characterized as CD3+ cells supporting the T cell mediated response to chronic MNV infection. Furthermore, MNV slightly increased expression of *Ifn*γ in ASF-colonized *Il10*^−/−^ mice, whereas in OMM^12^-assciated *Il10*^−/−^ mice, *Tnf*α was upregulated. In contrast, SFB co-colonization strongly upregulated expression of pro-inflammatory cytokines *Tnf*α, *Il1*β, and *Il12a*, but not *Ifn*γ in ASF-colonized mice 4 weeks post MNV infection. Expression of these cytokines was mainly unchanged in OMM^12^-associated mice. Discrepancies in *Il12a* and *Ifn*γ expression in ASF-colonized mice after SFB colonization suggest an IFNγ-independent production of IL12. Production of IL12 in an IFNγ-independent manner is hypothesized to be relevant for physiological and pathological immune responses (62). However, whether particular bacterial species play a role in downregulation of *Ifn*γ expression in chronic MNV infection needs to be further investigated. SFB colonization also stimulated expression of the regulatory cytokine *Tgf*β, while no increase in the expression of *Il17a* was observed in these mice. IL17A is a signature cytokine of Th17 cells, which can be stimulated by SFB. However, Th17 cells can acquire a regulatory phenotype and lose the *Il17a* expression signature, which is promoted by TGFβ (63). Furthermore, the presence of TGFβ, IL6, and IL1β supports differentiation of Th17 cells devoid of pathogenicity (64). In addition, a recent publication demonstrated that the epithelial cell endocytosis of SFB antigens is important for regulation of T cell homeostasis (65).

The intestinal epithelium is crucial for the maintenance of gut homeostasis (66). We previously showed that MNV induces intestinal barrier disruption by downregulating expression of TJ components, which regulate intestinal permeability (22). Moreover, altered expression and distribution of TJ proteins are accompanied by increased paracellular permeability which is associated with the development of the intestinal inflammation (67–69). Claudin 4 and 8 in particular are important for sealing TJs. In our study, MNV decreased expression of claudin 4 and 8 in mice colonized with ASF, but not OMM^12^, indicating that specific bacteria synergize with MNV to cause barrier dysfunction by influencing expression of TJ components. Furthermore, SFB colonization in mice carrying ASF prevented MNV-induced downregulation of these TJ proteins. However, SFB colonization did not influence their expression in OMM^12^-associated mice. Additionally, SFB co-colonization increased gene expression of *Muc2* and antimicrobial peptide *Reg3*γ in ASF-colonized mice. Mucin 2 is the main component of the mucus layer that keeps intestinal bacteria at a distance from the intestinal epithelium. The inner mucus layer contains various antimicrobial peptides produced by epithelial cells that exhibit bactericidal effects (70, 71). Colonization with SFB was shown to ameliorate colitis induced by the intestinal pathogen *Citrobacter rodentium* by increased expression of pro-inflammatory cytokines and antimicrobial defenses (31). Furthermore, several studies showed that animal models with an impaired MUC2 or REG3γ production pose an increased risk of developing intestinal inflammation (72–74).

As the phenotype of OMM^12^-colonized mice was not modulated by SFB colonization, we analyzed SFB colonization dynamics. We showed that SFB cannot colonize OMM^12^-associated mice and hypothesized that the absence of SFB-mediated protective effects in these mice is due to lack of SFB. Both the host immune response and intestinal microbiota itself regulate colonization processes in the gut. The host immune response can limit colonization by inducing secretion of cytokines and immunoglobulins (75). The intestinal microbiota confers colonization resistance mechanisms for protection against new and harmful organisms by competing for essential nutrients or secreting inhibitory bacteriocins (76, 77). Our results showed that SFB colonization depends on the presence of specific bacterial species. SFB could not colonize OMM^12^-associated B6-*Rag2*^−/−^ mice, excluding the adaptive immune response as a reason for unsuccessful SFB colonization. However, SFB could compete with OMM^12^ members when we reversed the order of colonization demonstrating that established OMM^12^ consortium prevents SFB colonization. The OMM^12^ consortium, despite its reduced complexity, provides partial colonization resistance to *Salmonella enterica* serovar Typhimurium infection (28). However, the exact mechanism of how OMM^12^ bacteria interfere with SFB colonization needs to be determined in future studies.

As MNV was shown to initiate barrier disruption in an early phase of infection, we analyzed whether SFB could prevent adverse effects of MNV in ASF-colonized B6-*Il10*^−/−^ mice 48 h post infection. In the acute phase of MNV infection, the presence of SFB strengthened the intestinal epithelium by increasing the production of antimicrobial peptides (ß-defensin 2 and REG3γ), MUC2 and tight junction proteins claudin 4 and claudin 8. Moreover, SFB modulated host immune response by upregulating expression of pro-inflammatory cytokines such as *Tnf*α, *Ifn*γ, and *Il1*β. However, the expression of the anti-inflammatory cytokine *Tgf*β, as well as *Foxp3*, a gene coding for a transcription factor involved in the development of Tregs, were also markedly upregulated in these mice. These results supported our conclusion that SFB can induce mucosal regulatory responses in the absence of IL10. Furthermore, type III interferons protect the epithelial barrier from viral infections and damage induced by bacteria, but also suppress intestinal inflammation in mice (78, 79). MNV did not strongly upregulate the expression of interferon lambda 2 (*Ifn*λ_2_) 48 h post infection, whereas ASF-SFB co-colonized mice showed higher expression of this cytokine. This suggests that upregulated *Ifn*λ_2_ expression is part of the SFB-mediated protective effect in the acute phase of MNV infection. Moreover, the presence of SFB resulted in the increased expression of *Mmp7*, which is predominantly induced by IFNλ_2_ (80). *Mmp7* is a member of the matrix metalloproteinase family of enzymes that are involved in tissue remodeling and wound repair. Altogether, our results suggested that SFB prevented development of inflammatory lesions in *Il10*^−/−^ mice by enhancing intestinal barrier defense mechanisms and inducing regulatory immune response. We showed that the severity of MNV-induced colitis in *Il10*^−/−^ mice depends on the intestinal microbial context and that SFB-mediated protective effects are multifaceted. Ultimately, it is essential to understand the fine-tuned interplay between the host and specific commensals, as this will deliver new strategies that can be used for disease interventions.

## Data Availability

The raw data supporting the conclusions of this manuscript are available from the corresponding author on reasonable request to any qualified researcher.

## Ethics Statement

This study was conducted in accordance with German animal protection law and with the European Directive 2010/63/EU. All experiments were approved by the Local Institutional Animal Care and Research Advisory committee and permitted by the Lower Saxony State Office for Consumer Protection and Food Safety (LAVES; file number: 14/1570 and 18A367).

## Author Contributions

AB, MBu, and MBa conceived experiments. AB and MBa supervised the study. SB, AS, MBa, DA, KO, and CE performed experiments. SB, CE, and MBa analyzed results. BS designed and provided Oligo-MM^12^. SB and MBa wrote the manuscript. MBa prepared figures. All authors reviewed the manuscript.

### Conflict of Interest Statement

The authors declare that the research was conducted in the absence of any commercial or financial relationships that could be construed as a potential conflict of interest.
